# Biotechnology and Solutions: Insect-Pest-Resistance Management for Improvement and Development of Bt Cotton (*Gossypium hirsutum* L.)

**DOI:** 10.3390/plants12234071

**Published:** 2023-12-04

**Authors:** Abdul Razzaq, Muhammad Mubashar Zafar, Arfan Ali, Pengtao Li, Fariha Qadir, Laviza Tuz Zahra, Fiza Shaukat, Abdul Hafeez Laghari, Youlu Yuan, Wankui Gong

**Affiliations:** 1 National Key Laboratory of Cotton Bio-Breeding and Integrated Utilization, Key Laboratory of Biological and Genetic Breeding of Cotton, Institute of Cotton Research, The Ministry of Agriculture, Chinese Academy of Agricultural Science, Anyang 455000, China; biolformanite@gmail.com (A.R.); m.mubasharzafar@gmail.com (M.M.Z.); 2 Institute of Molecular Biology and Biotechnology, The University of Lahore, Lahore 54500, Pakistan; fqadir935@gmail.com (F.Q.); lavizatuzzahra@gmail.com (L.T.Z.); 3 Department of Plant Breeding and Genetics, University of Agriculture, Faisalabad 38000, Pakistan; 4 Four Brothers Private Limited, Lahore 54500, Pakistan; arfan.alicemb@gmail.com; 5 School of Biotechnology and Food Engineering, Anyang Institute of Technology, Anyang 455000, China; 6 Center of Agri-Cultural Biochemistry and Biotechnology, University of Agriculture, Faisalabad 38000, Pakistan; fizaali.3855@gmail.com; 7 Department of Agronomy, Sindh Agriculture University Sub Campus Umerkot, Sindh 64470, Pakistan; ahlaghari@sau.edu.pk

**Keywords:** *Gossypium*, Bt cotton, biotic and abiotic stress, chemical insecticides, insects, sustainable cotton

## Abstract

Cotton (*Gossypium* spp. L.) is a major origin of natural fiber, and is projected at 117 million bales worldwide for 2021/22. A variety of biotic and abiotic stresses have considerable negative impacts on cotton. The significantly decreased applications of chemical insecticidal sprays in the agro-ecosystem have greatly affected the biodiversity and dynamics of primary and secondary insects. Various control measures were taken around the globe to increase production costs. Temperature, drought, and salinity, and biotic stresses such as bacteria, viruses, fungi, nematodes, insects, and mites cause substantial losses to cotton crops. Here, we summarize a number of biotic and abiotic stresses upsetting Bt cotton crop with present and future biotechnology solution strategies that include a refuge strategy, multi-gene pyramiding, the release of sterile insects, seed mixing, RNAi, CRISPR/Cas9, biotic signaling, and the use of bioagents. Surveillance of insect resistance, monitoring of grower compliance, and implementation of remedial actions can lead to the sustainable use of cotton across the globe.

## 1. Introduction

Plants are exposed to various biotic and abiotic stresses across their lifespan. Additionally, due to the current scenario of climatic change around the globe, the impact of these stresses has increased drastically, showing a remarkable influence on the yield of most crops [1]. Abiotic stress of, for instance, cold, salinity, high temperature, scarcity, heavy-metal noxiousness, and oxidative trauma are the major intimidations to the failure of crops in terms of growth and productivity, which could cause more than 50% of yield fatalities [2]. The seriousness of biotic stresses, not only cause losses in yield and low quality, but also increases the production costs due to the requirement for extra measures to be applied to control them [3].

**Figure 1:**
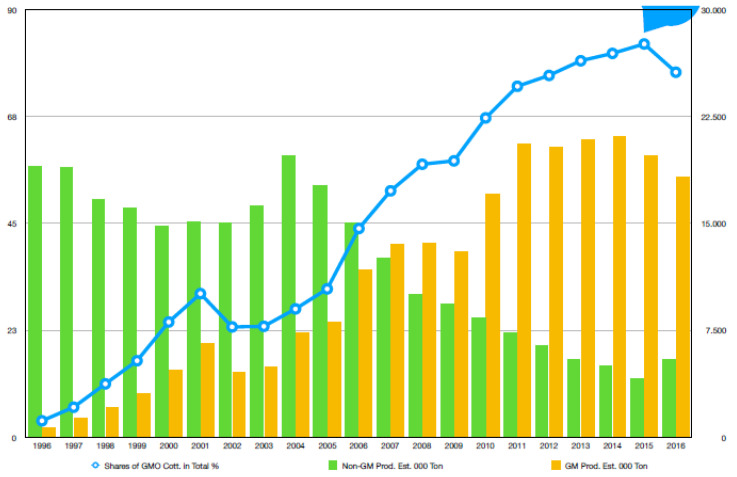
Global Bt- and non-Bt-cotton production and shares of Bt cotton.

Among all the available species of cotton, only four species are used for field production. Nevertheless, the highland cotton (*Gossypium hirsutum* L.) and sea-island cotton (*G. barbadense)*, belonging to plant family Malvaceae, are the major cultivated species grown in various areas of the world [4,5]. These two species contribute to 90% and 5% of the total worldwide cotton-planting acreages for fiber production in the world, respectively [6,7]. It is estimated that cotton is cultivated in an area of approximately 10,000 ha in excess of 80 countries per year [8], and 13% of which are in developed countries, while the remaining 87% are in developing countries where cotton is considered to be white gold [9]. A statistical data map of worldwide cotton-producing countries is shown in Figure 1.

The cotton plant generally possesses higher resistance to different abiotic stresses when compared with other crops [10]. However, during its life cycle, the plant could be subjected to various abiotic stresses and biotic stresses, including but not limited to pests [11].

Pest damage has long been a global problem in cotton production. Even though only a small fraction of insect pests has economic importance, the cotton plant may encounter an estimation of up to 1326 insect pests around the world throughout its whole growth season (www.cicr.gov.in). Extensive research has been conducted to investigate the prevalence of pests in cotton fields, primarily focusing on their economic impact on cotton yields [12,13]. Among the significant insect pests responsible for losses in cotton production are the cotton jassid (*Amrasca biguttula*), cotton aphid (*Aphis gossypii*), thrips (*Frankliniella schultzei*), spotted bollworm (*Earias vittela*), pink bollworm (*Pectinophora gossypiella*), American bollworm (*Helicoverpa armigera*), cotton mealy bug (*Phenacoccus solenopsis*), fall armyworm (*Spodoptera frugiperda*), and whitefly (*Trialeurodes vaporariorum*) [14,15]. These insect pests, along with diseases such as root rots, leaf blight, leaf spot, target spot, and verticillium wilt, contribute to economic losses ranging from 15% to 30% in cotton production, with some instances reaching up to 50%, which are attributed to direct damage or the transmission of plant diseases [14]. For instance, in Brazil, annual losses in agricultural production due to insect pests can average 7.7%, equivalent to approximately USD 17.7 billion [16].

The recent review summarizes the development and application of Bt cotton for over two decades; analyzes and discusses the impacts of Bt cotton on agro-ecosystems, and major issues relating to Bt-cotton production; and focuses on the current problems of Bt-cotton production, including the mechanisms of evolution of pest tolerance against Bt cotton, for the purpose of improving Bt efficacy for better control strategies of the target pests to ensure cotton-production stability in the future. First, we briefly review the perception and implementation of Bt cotton in production; then, we discuss the dynamics of bio-communities in Bt-cotton fields, including the target, non-target pests, and the bio-communities; third, we examine the evolution of pest resistance to Bt toxin (PRBT); and, finally, we deliberate approaches to improve the efficacies of Bt resistance and control against its target pests and to delay the development of pest tolerance to Bt toxin. We believe this review will help and promote the future research and development of Bt cotton and pest-control management.

## 2. Global Perception and Adoption of Bt Cotton for Better Yield and Pest Control

Due to the potential limitations of conventional strategies for pest management in cotton production, genetically engineered cotton expressing the insect-specific Bt-toxin proteins, from the naturally occurring soil-born bacterium *B. thuringiensis* (Bt), was applied in cotton production. The insecticidal properties of Bt toxins from Bt have been recognized by human society for a long time even dated to early in ancient Egypt, while the isolation of Bt strains and their use in pest control started in the beginning of the 20th century [14,17]. For detailed information regarding the identification, the research activities of these bacteria, and the properties and applications of the different Bt toxins please see the review paper of Sanahuja et al. [17]. To date, almost 100 different subspecies of *B. thuringiensis* have been identified which produce the insecticidal toxin named Cry, Cyt, or Vip [18]. Cotton bollworms are susceptible to *Cry1Ac* and *Cry2Ab* toxins, while the corn borer is highly sensitive to *Cry1Ab* and the corn rootworm to *Cry3Bb* toxin. The *cry* gene was initially incorporated into tomato and tobacco genomes for the control of *Lepidopteran* insect pests and subsequently in other major crops including cotton, corn, soybean, and rice, etc. [19]. After the importance of the insect-pest problem was realized in cotton production, Bt cotton was adopted by major cotton-producing countries at different times.

The first commercial release of Bt cotton took place in the USA in 1996, and was conducted by Monsanto in collaboration with Delta and Pineland Company (D and PL). In the following years, Bt-cotton-planting acreage increased rapidly, occupying 15% in 1997, 37% in 2001, and 85% in 2019 of the total cotton-production areas in the USA due to its performance of increasing cotton yields, while reducing pesticide applications. This highly dramatic adoption rate of Bt cotton in the USA ensued a glaring reduction of pesticide costs, enhanced resistance of Bt-cotton crop against tobacco budworms, cotton bollworms, and pink bollworms, and, finally, resulted in significant profitability [11].

China has made a large investment in biotechnology, which was accelerated in the late years of the 1980s by the “863 project” initialed by the Ministry of Science and Technology of China. Due to an immense pressure of the problem of the insect pests in the northern region of the cotton-planting area in China (Huanghuaihai Valley Region), scientists in the Chinese Academy of Agricultural Sciences worked on plant transformation techniques and started to look at this as a promising tool of introducing Bt *cry* genes (*cry1Ab* and *cry1Ac*) into cotton genomes to combat this severe insect-pest problem. In the mid-1990s, Monsanto together with D and PL started to collaborate with local Chinese companies to introduce some Bt-cotton cultivars on a commercial scale into the Chinese market. In fact, in 1996 Bt cotton was approved for commercial release in the market by the Chinese Biosafety Committee [20]. Afterwards, Bt-cotton acreage also grew rapidly and steadily in China, and it was estimated that Bt-cotton-planting acreage rapidly increased from 1.5 million hectares to a total of 3.5 million hectares in 2001. In a five-year period of survey, the rate of adoption of Bt cotton in China was extremely high due to its increased yield per ha, reduced pesticide costs and incidences of pesticide poisoning, and better pest control [21,22].

Pakistan is an agricultural country and ranks 4th among the major cotton producers worldwide. The perception and adoption of Bt cotton experienced a problematic period in terms of safety, quality, yield, and effectiveness against insect pests [23]. Initially, the Government of Pakistan approved the use of Bt cotton on a trial basis in different cotton-growing areas therein. A survey conducted in 2009 in the major cotton-growing province of Punjab showed that, at the farmer level, Bt cotton was more effective and promising than non-Bt cotton [23,24] even though there were still attacks of some insect pests such as cotton bollworms (*Helicoverpa armigera*), pink bollworm (*Pectinophora gossypiella*), spotted bollworms (*Earias vittella* and *E. insulana*), tobacco cutworm (*Spodoptera litura*), beet armyworms (*S*. *exigua*), and some sucking insects (*Bemisia tabaci*, *Thrips tabaci*, *Amrasca devastans* and *Aphis gossypii*), as well as high incidence of cotton leaf curl virus (CLCV). The benefits of Bt-cotton planting included significant reductions in total pesticide application for pest control, and, in labor input, higher yield and cost efficiency. But at that time the farmers still used to use chemicals to ensure effective control of insect-pest attacks. This showed some farmers’ reluctance to adopt Bt cotton [25,26]. After some time, however, Bt-cotton-planting acreage increased steadily, with some traditional wheat and sugarcane growing areas being shifted to planting Bt cotton. A long-term observational study from 2003 to 2013 revealed that, of the two main cotton-producing regions, Punjab had a much higher adoption rate of Bt cotton than Sindh [27].

In India, cotton is also a major crop. The major efforts to use Bt cotton to harness the damage of the bollworm started in the late 1990s with the import of Bt cotton from Monsanto. The initial field trials of Bt cotton showed 40% more yield with 50% chemical insecticide reduction of Bt cotton compared to non-Bt cotton [28]. In February 2002, the Indian Council of Agricultural Research (ICAR), Ministry of Environment, and the Genetic Engineering and Approval Committee mutually approved three Bt-hybrid cottons MECH 162-Bt, MECH 184-Bt, and MECH 12-Bt, developed by Monsanto for commercial distribution. Soon after the approval, these hybrids were planted in six different Indian states, Andhra Pradesh, Gujarat, Karnataka, Madhya Pradesh, Maharashtra, and Tamil Nadu [29,30]. Although, that year experienced unfavorable conditions for overall cotton production due to heavy rainfall followed by dry weather, which led to a very low pest pressure, and a significant decline in chemical sprays was observed, indicating a substantial effect of Bt cotton in comparison to non-Bt cotton on the control of the bollworm complex [29]. In the following seasons, Bt-cotton-planting acreages rapidly increased from 0.56 million ha in 2004/5 to 3.7 million ha in 2006/7. It was estimated that during the period from 2001 to 2011, the production of Bt hybrid cotton increased from 1.56 million bales to 3.56 million bales [28]. Then, the Cotton Advisory Board of India (CAB) reported a significant decline of acreage of Bt cotton from 95% to below 90% during the period of 2013/14–2016/17 due to the stagnant yield of Bt cotton and the increased attacks of pink bollworm in these Bt-cotton fields. The attacks infected an acreage of 8.77 million ha of the total 10.82 million ha of cotton-production acreages therein. This decline stopped in 2017, and the Bt-cotton percentage recovered in the year 2017/18 by 8%, which indicated a high adoption rate of Bt cotton in India [31].

Cotton is grown in different states of the USA. The insect-resistant Bt cotton containing Cry1Ac was commercially introduced into the market by Monsanto. The adoption and cultivation of Bt cotton eradicated the detrimental insect–cotton boll weevi and significantly decreased the use of pesticides. With this success later on, both *Cry1Ac* and *Cp4-EPSPS* genes containing cotton were successfully introduced into the market [32]. Since 2000, Brazil has become one of the leading countries in agricultural production. Brazil produces different economic crops such as sugarcane, soyabean, coffee, corn, and cotton. During the 1970s−1980s, cotton was grown on small farms in the cerrado province of Brazil, but it was affected by the emergence of cotton boll weevi. Later on in the 1990s, transgenic cotton was adopted on a large scale. Since then, the Bt-cotton production has increased in Brazil [33]. The cultivation of cotton has increased in Turkey since the 1990s. Cotton is grown in different regions of Turkey like Antalya and Anatolia especially in the Aegean regions. Turkey is a pioneer in producing natural-colored and organic cotton around the globe because it has not applied transgenic technology to its cotton production [34]. Bt cotton was commercialized for the first time in Mexico during 1996. After the adoption of Bt cotton in 2008, the 96% cotton field was planted with Bt cotton. In Argentina, after the adoption of the transgenic soyabean, Bt cotton was adopted and cultivated in Argentina. Firstly, Bt cotton was adopted by small farms and families. The cultivation significantly reduced the cost of pesticides and increased the output. Argentina is now one of the global leaders in Bt-cotton production [35].

## 3. Effect of Bt-Cotton Fields on Bio-Community Interactions

### 3.1. Effect on Target Pest

**Figure 2:**
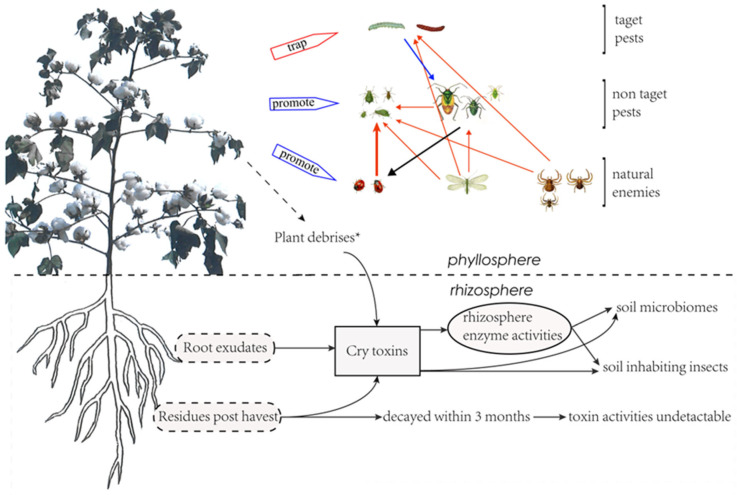
Changes in pest-community interactions due to Bt cotton and Bt toxins. ^★^ Plant debrises include defoliation, pollen falling, and sqare and boll shedding.

Cotton is the main host crop of cotton bollworms. From June to July each year, cotton bollworms (*H. armigera*) migrate to the cotton field and lay their eggs around the flower organs [36]. The hatched larvae feed on the young tissues and thus cause damage to the plant. The toxin protein in Bt cotton kills the feeding larvae, which makes it impossible for cotton bollworms to complete their life cycles. Thus, the density of eggs and larvae of the cotton bollworm steadily decreased, along with the increase in planting years and proportion of Bt cotton, as revealed in monitoring from 1992 to 2006 in China [37]. The large-scale planting of Bt cotton not only effectively controls the damages of the cotton bollworm to cotton, but also makes the Bt-cotton field a deathtrap for cotton bollworms in the agricultural ecosystem (Figure 2), in which cotton serves not only as a major host of second-generation cotton bollworms, but also as core source of third-generation cotton bollworms. This trap can break the host chains of cotton bollworms, making their threats to other host crops including corn, peanut, and soybean gradually decrease [36,37,38]. The fact that the severity of the cotton bollworm in corn, peanut, sunflower, and other crops increased significantly, along with the reduction of the Bt-cotton-planting scale in Yellow River Valley after 2010, confirmed the “death trap” effect of the Bt field.

Pink bollworms *(P. gossypiella)* are another major pest of cotton plants, which mainly attack the flowers, buds, developing bolls, and seeds, leading to a significant decrease in yield and lint quality [22,39]. They also attack other plants including kapoks and mallows, while in agricultural ecosystems, comparatively, the cotton plant is their exclusive host. This characteristic makes the population of this pest decrease rapidly along with the expansion of planting acreage of Bt cotton [40]. Thus, when Bt cotton was under an integrated pest-management (IPM) framework, the regional catastrophic risks of target pests and some other sporadic pests in cotton fields are brought under effective control at significant cost due to pesticide sprays [41,42].

### 3.2. Effect of Bt Cotton on Non-Targeted Pests

Bt-cotton planting has no significant effect on the individual development and population dynamics of non-target pests or natural predators, but is a strong indicator of the reduction in pesticide spray [42,43]. The reduction of pesticide sprays in Bt-cotton fields resulted in the enhancement of the biodiversity of insect species, including the population dynamics of non-targeted pests and their natural predators [36], and the interaction between them. It was observed in a study that the populations of the three main natural predators in cotton fields were ladybirds, lacewings (*Chrysopa perla*), and spiders, whom significantly increased along with Bt-cotton-plant scale from 1990 to 2010. Their predation behaviors were also enhanced and significantly decreased the natural populations of their prey, pest aphids, in Bt-cotton fields [36] (Figure 2). When these predators migrated to the fields of corn, peanuts, and soybeans, they also played a significant role in the natural control of aphid populations in these fields [44]. The second significant impact of reduced sprays in Bt field is that it allows the non-target secondary pests to become a major problem. The typical example is of mirid bugs, which are usually considered a secondary pest in cotton fields [44,45]. Mirid bugs have a habit of gathering in areas adjacent to the flowers and buds of host plants. At the flowering stage of the cotton plant, they coincidently migrate to the cotton field with the cotton bollworms, and this leaves them lethally trapped by the chemical sprays that control cotton bollworms. Therefore, in a conventional cotton field, controlling mirid bugs is usually not taken into consideration when primary pests are present. The predating behavior of natural enemies in the cotton field has little impact on the population development of mirid bugs [42,46]. Thus, decreased spraying of agrochemicals in Bt-cotton fields favors the development of the mirid bug population and eventually promotes them to become primary pests [17,44,47]. The outbreak of mirid bugs may bring arthropod pests under suppression [47] (Figure 2).

### 3.3. Effect of Bt-cotton Residues on Soil Bio-Communities

The constitutive expression of the Bt toxin in the transgenic Bt-cotton plants and the water-soluble property of the Bt toxin [43,48,49] may lead to concerns of Bt-toxin persistence in soil. The presence of *cry*-gene residues could be associated with the large-scale release of Bt-crops. Usually, there are plenty of leaves, stalks, roots, and falling bolls left in the Bt-cotton field after harvest, and it may take several months for these residues to decompose. The concerns might include two aspects, one is the persistence of these Bt-toxin-protein and *cry*-gene residues in the field and their dynamics therein, and the second is the impacts of these residues on agricultural ecosystems. Thus, studies have been conducted both in controlled laboratory conditions and in open field environments [14,50]. Tests of soil samples taken consecutively outside and inside Bt-cotton fields after three to six years of Bt planting indicated that three months after harvest the soil samples contained extremely low levels of Bt toxin, resulting in no detectable biological activities [50,51]. However, when the soils were sampled three months after the last season’s tillage, which was conducted with shredders and disk plowings, most of the plant residues were found decayed. The dynamics of the Bt toxin in those plant residues and its biological activity were unclear. Results from Bt-rice and Bt-corn suggested no significant differences in the soil’s neutral phosphatase activity [52], and there was no noteworthy variations in the decomposition of Bt-corn residues and the composition of organism communities in the soil [53]. In a controlled assay, about 50% of the introduced Bt toxin persisted in soil for at least 56 days; the activities of soil urease, acid phosphomonoesterase, and invertase were inspired, while that of soil arylsulfatase was inhibited [54]. Long-term studies indicated that consecutive lodging of Bt cotton might lead to the persistence of the Bt toxin in soil [55] and impose a negative impact on soil microbial and biochemical properties [56]. These controversial results indicate that it is still essential to evaluate the lasting impact of Bt crops in various circumstances.

## 4. Abiotic and Biotic Constraints to Bt-Cotton Production

### 4.1. Temperature

Unstable resistance of Bt cotton to insect pests has been observed during the boll-development stage, particularly under prolonged high-temperature stresses [57]. Low relative humidity together with high-temperature conditions contribute greatly to the reduction of Bt toxin content in Bt cotton leaves [57,58]. It was also observed that leaf Bt-toxin content was negatively correlated with leaf C/N ratio, which was enhanced by high temperature and nitrogen levels [59]. High-temperature stress had a similar impact on the Bt-toxin concentration in Bt-cotton plants [60]. The reduction of Bt-toxin content was thus responsible for the observed fluctuation of insect-pest-control efficacy under high-temperature conditions.

### 4.2. Drought

An estimation of about 30–50% annual crop loss worldwide was due to environmental stresses [61], and drought is considered as a substantial factor for crop-productivity losses [62].

Under increased drought stress, the contents of Bt toxins like Cry1Ac and Cry2Ab in Bt cotton declined, and the condition recovery was correlated to a pronounced increase in Bt-toxin content, indicating an instability of Bt-toxin production under abiotic stresses [63]. In addition, a study based on a collection of 32 cotton cultivars revealed that the Bt-toxin-content decline was correlated to the crop resistance to bollworms under drought-stress conditions [64].

### 4.3. Salinity

High salinity is a major threat to agricultural lands leading to land degradation, soil biology disturbance [65], growth retardations [66], yield losses, and quality attributes of cotton fibers globally. Sodium and chlorides are the most important ions contributing to soil-salinity-inducing plant disorders [67].

To remediate salt-affected soils, cotton has been planted in saline or coastal areas in Eastern China as a pilot crop for soil rehabilitation. The growth and development of *H. armigera* was studied in Bt-cotton fields under such conditions [68]. The results revealed that no significant difference was observed for the growth and development of *H. armigera* larvae under certain content ranges of saline conditions, but high-salt stress altered the larval growth and development, and adult oviposition behavior on Bt cotton [68]. This result provides some insights for the pest management of *H*. *armigera* for Bt cotton in saline-soil conditions.

### 4.4. Climate Change

In this century, the growing concentration of atmospheric CO_2_ and rising temperatures are the major challenges of global climate change. The forecast is of increased CO_2_ from the current 400 ppm to between 750 and 1300 ppm by the end of this century, while, in the same period, the global mean surface air temperature is anticipated to rise about 1.8–6.0 °C (IPCC, 2014). The resistance mechanisms of cotton insect pests in the context of climate change are not well studied. The elevated CO_2_ leads to effector-triggered immunity (ETI) and PAMP-triggered immunity (PTI) defenses [69]. Autumn is neglected due to climate change, and this causes alteration in insect reproduction rate, migration, and induction of diapause [70]. The genetic adaption to the changing climate is necessary to avoid population extinction, but genetic changes due to climate change are not well documented [71]. Studies on physiological responses of subsequent generations of insects exposed to increasing CO_2_ and temperature are rare [72].

The outcomes of O_3_ on leaf nutritional quality are not well studied but higher leaf senescence lowers the quality for insect herbivores, while alteration in the secondary chemistry and microclimate of leaves under elevated CO_2_ and O_3_ makes the plants more susceptible to insect herbivores [73,74,75]. The invasion of the fall army worm occurs only in those regions that have similar climatic conditions to the native distribution [76]. In applied ecology, the breakout of alien insects to a new land climate is an emerging issue. If evolutionary alteration of their life-history characters is fairly rapid, we cannot construct superficial forecasting for their invasion based on the properties in their original lands [77]. Climate change has increased resistance against insecticide in *P*. *solenopsis* due to the increased number of generations and shorter life cycles [78].

#### 4.4.1. High Carbon Dioxide (CO_2_)

Due to elevated CO_2_, the plants C/N ratio is increased which effects the C-based secondary chemistry. The plants’ nutritional level, reduced because of these changes, results in low nitrogen concentration and high phenolics [79]. The raised CO_2_ can have diverse special effects on various trophic levels, plants, herbivores, and predators/parasitoids [80]. The elevated CO_2_ (E-CO_2)_ effects the behavior of larval feeding and enhances the developmental time. It also causes reduction in adult weight, survival, and fecundity of insect herbivores, as well as altering insects’ antioxidant capabilities [81]. The chewing insects in E-CO_2_ grow slowly with a higher consumption and mortality rate [82]. Their fecundity is also reduced (e.g., cotton bollworm) under elevated CO_2,_ but the number of offspring has increased in the case of aphids [83]. The larval growth of the fifth and sixth instars of *H*. *armigera* is slower with enriched CO_2_ as compared to ambient CO_2_. The female pupal weight was also lower with enriched CO_2,_ but the duration of the pupal stage was not affected. The enriched CO_2_ had an adverse effect on the growth and fitness of *H*. *armigera* [84]. The consumption and metabolic rate is higher in *H*. *armigera* due to increased protease activity and carbohydrates under elevated CO_2_ and temperature [85]. The damage may increase because of a higher consumption rate under elevated CO_2_ [86]. The concentration of Bt toxin was reduced, but the concentration of gossypol, terpenoids, phenolics, and condensed tannins were increased in cotton under E-CO_2_ [87]. Under E-CO_2,_ the plant response is weaker to the attack of insect herbivores [88]. The insects compensate for the nutritional deficiency due to N content dilution under E-CO_2_ through increasing their food intake, which causes severe damage to the host plant [89]. The E-CO_2_ increases the fecundity of the cotton aphid [85]. The aphid responses are species specific to E-CO_2_ and were the only feeding guild to respond positively to E-CO_2_ [90]. In future, elevated CO_2_ application of nitrogen fertilizer to maintain C-N balance in transgenic plants is an attractive approach [87].

#### 4.4.2. Imminent Temperature

The prominent raised temperature caused an acceleration in insect growth, a decreased period of cohorts and productiveness, extends the dissemination of insect inhabitants, and also encourages some functional responses [91]. The effect of E-CO_2_ may be concurrently aggravated or alleviated in insects at a prominent high temperature [80]. The positive effect of E-CO_2_ on aphid performance is counteracted by a high temperature. In *Bemisia tabaci*, the eminent temperature and E-CO_2_ expressively enhanced GST and AChE expression in the first cohort group, CAT action in the third generation, and lower SOD expression in the third generation [92]. The manganese superoxide dismutase (MnSOD), peroxidase sulfate (PODS), catalase (CAT), acetylcholinesterase (AChE), and glutathione-S-transferases (GST) are important antioxidative enzymes in insects [93,94]. The protection enzymes are SOD, CAT, and POD, whereas the detoxification enzymes are GST and AChE in insect herbivores. The SOD activity is increased under E-CO_2_ in *H. armigera* [82] and *A. gossypii* [95].

The fluctuation in temperature influences the infestation of whitefly and jassid as compared to other environmental factors [96]. The temperature affects the activity of P450 in whitefly which ultimately influences the tolerance of whitefly to insecticides. The activity of *CYP6CM1* is significantly upregulated in whitefly at 31 °C and suppressed at 35 °C [97]. The cotton jassid population is positively correlated with high temperature and negatively with low temperature [98]. The invasion of the fall army worm (FAW) occurs only to those regions that have similar climatic conditions to the native distribution [76].

Prominent CO_2_ and temperature enhance the consumption of food and the metabolism of larvae by increasing the activity of midgut proteases, carbohydrase’s (amylase and cellulase), and mitochondrial enzymes, and, therefore, may cause more damage to crop production. The growth and development of an insect is affected by elevated CO_2_ and global warming, which alters the pests and host-plant interaction [99]. Climate change also alters the insect genetics, invasion, and number of generations; therefore, there is a dire need to expand the understanding of these interaction to develop strategies to mitigate the upshots of climate variation.

### 4.5. Diseases

Cotton produces substantial economic return for approximately 150 countries with a planting acreage of 33 million acres providing income for approximately 100 million families [11]. It was estimated that more than 40 different diseases caused by bacteria, viruses, fungi, and nematodes have been reported on cotton plants [100] causing 10–30% annual-yield loss of cotton production worldwide. *Xanthomonas citri* pv. *Malvacearum* is a major pathogen that causes bacterial blight in *G*. *hirsutum* [101]. Fusarium wilt caused by *Fusarium oxysporum* f. sp. *vasinfectum* [102] and Verticillium wilt caused by *Verticillium dahliae* are two major fungal pathogenic diseases [100]. In its whole life cycle, the cotton plant may also suffer from an attack of anthracnose (*Colletotrichum gossypii*), ramulosis (*Colletotrichum gossypii* var. *cephalosporioides*) [103], ramularia gray mildew (*Mycosphaerella areola*) [104], root rots (*Sclerotium rolfsii* and *Rhizoctonia solani*) [105], leaf blight (*Alternaria macrospora*) [106], leaf spot (*Cercospora gossypina*) [107], and target spot (*Corynespora cassiicola*). As, currently, Bt cotton occupies the absolute majority share of the cotton-planting market, how Bt cotton responds to the diseases’ stress represents the stability of cotton production. However, the effects of these diseases on Bt cotton are still kept open to discussion, especially the stability of Bt protein expression.

### 4.6. Weeds

Weeds compete with crops for the available resources, such as sunlight, water, nutrients, and space, all for their growth and development. They also provide spaces and shelters for plant pathogens and pests, which may interfere with plant growth and development. Different weed-controlling strategies such as mechanical (such as manual hoeing), and chemical (such as herbicides) are adopted as integrated weed management [108,109]. Planting Bt cotton may not significantly increase the weed diversities or the risks of producing newly destructive weed species. Spraying herbicides together with tillage practices could be effective for weed managements in both conventional and Bt-cotton fields. When studied in natural wild habitats, Bt- or non-Bt-cotton seeds did not differ in their ability to germinate, establish, and survive, demonstrating that the addition of the Bt gene does not confer fitness for weeds or establish invasive cotton populations [110,111].

## 5. Evolution of Pest Resistance to Bt Toxins

Although the extensive cultivation of Bt cotton around the globe has brought benefits such as pest-control efficacies and economic preferences, it also imposes strong selection pressure on the target cotton pests, which has facilitated the evolution of target-pest resistance to Bt toxin (PRBT), thereby reducing its efficacy [112,113]. After over two decades’ of application of Bt cotton, the potential threats of the resistance development of bollworm to Bt toxins are increasing. This issue was recognized as a potential problem as early as the beginning of twentieth century [6]. Various studies have recorded this threat due to irregular expression of the toxin in host plants. Many studies were then conducted to survey the resistance development of pests against Bt toxins in various selected insects under laboratory or field conditions [114]. Accumulative records of the evolution of PRBT were observed in Bt-cotton fields in China [115], India [116], the USA [117], and in various geographical regions of the world, including South Africa [118], Argentina, and Brazil [119]. The documentations of the rapid evolution of PRBT in Bt crops [120,121,122,123] have encouraged researchers to attempt to understand the genetic basis of PRBT in order to develop alternative counter mechanisms.

Studies revealed that multiple mechanisms are involved in the resistance to the evolution of pests of Bt crops, including variations in toxin activation, mutations in toxin receptors, and regulation of immune systems, and for details of which please refer to the review of Xiao and Wu [113]. In the current review, we examine factors that contribute greatly to the evolution of PRBT in Bt-cotton fields. These factors include lack of regulation and/or compliance with the Environmental Protection Agency (EPA) policies, sub-lethal expression of Bt genes in plant tissues, continuous exposure to the same Bt toxins, and cross resistance to multiple Bt toxins [124].

The long-term practice of planting Bt cotton has led countries to include a variety of complex integrated governance systems to manage the PRBT evolution, and incompliances with these resistance-managing measurements will lead to the failure to manage PRBT evolution [113,125]. A recent study on the environmental and agronomic impacts of Bt cotton in Mexico over the past 20 years has shown that the management strategies used there to prevent PRBT evolution in cotton fields are successful, and no adverse effects on non-target organisms have been observed [126]. Similar strategies are recommended in other regions where Bt cotton is planted for cotton production.

The sub-lethal expression of *cry*-genes in plant tissues is one of the principal causes of the evolution of bollworm resistance to Bt toxins [127]. Actually, the expression of the insecticidal Bt toxins in Bt cotton is inconsistent in different genotypes [128], occurring in different parts and tissues of plants [96], showing a decreasing expression pattern as plant-age advances [129]. The factors that were reported to be responsible for low expression of *cry* genes were summarized in Table 1. Cross resistance could also lead to the evolution of PRBT. In a pyramiding strategy, transgenic cotton producing the two Bt toxins Cry1Ac and Cry2Ab was used to control *Helicoverpa zea*, a major pest in North America; it was demonstrated that selection with Cry1Ac increased the pest survival on cotton plants expressing the two toxins of Cry1Ac and Cry1Ab. Further analysis indicated typical cross-resistances between Cry1A and Cry2A toxins [130,131]. Cross resistance of *H. zea* to some Bt toxins has also been documented in some other pyramiding events [132,133].

Continuous exposure to the same Bt toxins in a large-scale Bt crop will generate a large selective pressure for pest resistance, especially in the laboratory conditions, where rapid evolution of PRBT was observed when target pests are exposed continually to the same Bt toxins. The laboratory results may not represent the actual happenings in the field, but they provide early warning of potential resistance problems [138]. Long-term monitoring of Bt-cotton planting in China revealed that the sensitivity of field colonies of the cotton bollworm to Cry1Ac toxin decreased under the continuous exposure pressures; although, no failure cases of Bt cotton control were recorded [139]. A study of Tabashnik and colleagues [140] showed that the frequency of resistance alleles had increased substantially in some field populations of *H. zea*. Although this statement caused some debates [138], evidence of PRBT evolution gradually accumulated. Practically, resistance was observed in the insect species of *B. Fusca*, *D.v. virgifera*, *H. zea*, *P. gossiypiella,* and *S. frugiperda* against *Cry1Ab*, *Cry3Bb*, *Cry1Ac*, *Cry1Ac*, and *Cry1Fa*, respectively [141]. Fall army worm (*S. frugiperda*) has developed a maximum level of resistance against *Cry1Ac*, *Cry2Ab*, and *Cry1Fa* [142].

## 6. Strategies to Tackle Problems of PRBT Evolution

The emergence of resistance to Bt toxins in pest populations has prompted an immense interest of scientists to investigate the resistance mechanisms and to propose strategies to effectively manage the pest damages in Bt-cotton fields for sustainable cotton production. The strategies include phenotypic plasticity, investigation of individual *cry*-gene resistance in specific plant species for specific insect control [130], refuge crop strategy [143], mixing of seeds harboring different toxin genes, stacking two or more insecticidal toxin genes for one target insect [144], releasing sterile insects [145], bio-control agents, and bio-signaling [144]. Furthermore, whole-genome sequencing of the pest genome enabled researchers to identify genetic variations and QTLs using molecular markers including SSR, SNPs, and InDels to predict genetic interactions between pests and the host crop [146]. The advent of clustered regularly interspaced short palindromic repeats and caspase 9 activity (CRISPR/Cas9) [147], coupled with the above-mentioned strategies, prompted scientists to develop integrated strategies for the management of PRBT evolution to slow down the pace of advent of resistant pests of Bt cotton.

### 6.1. Phenotypic Plasticity

It is hypothesized that resistance development is not only a genetically controlled phenomenon, but also that of gene expression which reveals the impacts of environmental interactions [148]. Phenotypic variations of a trait depend upon the expression of other genes. A single genotype (individual gene) may be responsible for multiple phenotypes in different environments [149]. Phenotypes of a trait controlled by a gene or multiple genes were greatly influenced by macro- and micro-environments. The role of the environment in the resistance performance of Bt crops has been neglected. In the case of Bt cotton and for bollworm control, a Bt cultivar could exhibit high resistance to bollworm (implying high susceptibility of the insect to Bt toxin produced by the plant) in one environment, while low resistance (low susceptibility of the insect) is present in the other, suggesting phenotypic plasticity of Bt cotton [148]. For pink bollworms, their monophagy nature encourages them to feed on their preferred diet. This strict diet of pink bollworms may regulate their susceptibility and resistance to Cry proteins [150]. In a laboratory examination, when the larvae of corn earworm were fed with different nutrient combinations of a carbohydrate–protein diet, they showed variations in survival when challenged with Cry1Ac protein [148].

### 6.2. High-Dose/Refuge Strategy

High-dose/refuge strategy was probably the first worthy strategy in consideration for the management of PRBT evolution that was put into research and practical application. However, as mentioned above, the success of dose strategy requires a high concentration of the Bt toxins expressed in the plant which ensures ≥95% mortality of the heterozygous pest individuals that have one copy of the resistance allele. According to the U.S. Environmental Protection Agency, a dose 50 times higher than the concentration for killing 50% of Bt-susceptible larvae is required to assure the success of the high dose/refuge strategy. In this strategy, high expression of the *cry* gene reaching consistent lethal levels in all plant tissues on which the pests feed is indispensable for effective pest control [116,128]. One important fact of this high-dose strategy for management of PRBT evolution is that the dose concentration that ensures effective resistance against one target pest may not be effective against another [151]. The refuge strategies are based on three assumptions: recessive-resistant mutation to Bt toxin, low frequency of resistant mutation, and effective dilution of resistant mutations in susceptible populations which are planted near the Bt crop [113]. Most of the resistant mutations are recessive ones that must be homozygous to display phenotypes [132,152,153,154]. Then, refuge arrangement and layout in Bt-crop fields for the particular insect pest are critical for the success of a refuge strategy for the management of PRBT evolution. The effective refuges can be conventional non-Bt-cotton plants, other field crops including corn, peanut, soybean, and vegetables [143]. In the case of using different host crops of a pest species as refuges for the management of PRBT evolution, the crops that were introduced with similar Bt genes cannot be used as refuges to each other, because such crops may share a common resistance selection to the target pests [144]. This circumstance is observed in parts of the USA Cotton Belt where common hosts of *H. zea*, Bt corn, and cotton are planted, and in the Cerrado region of Brazil where the fall armyworm and *Helicoverpa* spp. are hosted by Bt corn, cotton, and soybeans [125]. The refuge strategies provide enough susceptible insects to effectively dilute the pests with resistant locus to delay the accumulation of resistant-pest population. In a four-year field study on *H. armigera* against Bt cotton expressing Cry1Ac toxin in six provinces of China, the results revealed an increase in the percentage of resistant insects from 0.93% in 2010 to 5.5% in 2013 [143], much lower than the model prediction of 98% in the same time spell without natural refuges. The results suggest that the natural refuge strategy effectively delays resistance development in the pest [143].

Mixing some non-Bt seeds into Bt seeds was found to be an effective method of dose strategy for the management of PRBT evolution in transgenic Bt-cotton fields. This methodology ensures that growers comply with the refuge strategy by prior mixing the Bt and non-Bt seeds. In an 11-year study in China, transgenic Bt seeds/plants were crossed/mixed with conventional non-Bt cotton seeds/plants, with the second filial generation (F_2_) consisting of 3/4 Bt plants producing Bt toxin, and 1/4 of non-Bt plants were planted to perform the study. The results demonstrated that the seed-mixing strategy effectively delayed the resistance of the pink bollworm (*P. gossypiella*) against the Cry1Ac toxin [155].

### 6.3. Release of Sterile Pests

Release of sterile pests in the Bt-cotton field demonstrated a significant delay in the development of resistant pink bollworm to Bt toxins [145,156]. Sterile insects were released to mate with the resistant pests under the condition of there being no refuges in Arizona. Computer simulations show that this method works effectively against pests with recessive or dominant inheritance of resistances. Over a 4-year period and large-scale adoption of this strategy, the resistance of *P. gossypiella* against Bt cotton did not increase [145]. When this ‘release of sterile pest’ strategy was incorporated into a multitactic eradicating program, the abundance of *P. gossypiella* in a Bt-cotton field was reduced by >99%, showing its effectiveness in delaying the development of resistant pink bollworm to Bt toxins [145].

### 6.4. Stacking of Genes and RNAi

A gene-stacking strategy, expressing two or more different insecticidal toxins in the transgenic plants against the same target pest, was proven to be highly effective in delaying resistance development [124]. In 2003 and 2006, the two *Cry* genes *Cry2Ab* and *Cry1Fa* were, respectively, stacked with the *Cry1Ac* gene and introduced into the cotton genomes. Then, Bt cotton plants that produce only the Cry1Ac toxin were replaced with the newly engineered Bt plants that produce two Bt toxins, Cry1Ac + Cry2Ab or Cry1Ac + Cry1Fa, for the control of *H. zea* [130,131]. The results show that such a gene-pyramiding strategy can effectively ameliorate the management of PRBT evolution. Although it does not eliminate the need for implementing strategies as ‘high-dose ⁄refuge’, it really alleviates some of the selecting pressure of the latter [124].

An attempt to stack RNAi strategy to Bt cotton has also been proved to be effective in slowing down the resistance development of the pests. In this strategy, a double-stranded RNA construct was introduced into cotton genomes, targeting the P450 monooxygenase gene, *cyp6AE14*, an important gene in bollworm metabolism, which enables *H. armigera* to digest a diet containing gossypol. When larvae were fed with such transgenic plant tissues expressing this double-stranded RNA construct, the transcription of this key *cypP6AE14* gene in midgut cells of the pests was silenced, leading to retarded larvae growth due to the gossypol intoxication produced by the cotton plants [157]. When two RNAi constructs targeting *H. armigera* metabolism genes, the juvenile hormone acid methyltransferase (JHAMT) gene and the juvenile hormone binding protein (JHBP) gene were stacked with a *Bt* gene in cotton plants, respectively, in China, the pyramided cotton combining a Bt toxin and RNAi substantially delayed the resistance evolution in pests compared with using Bt cotton alone [158]. However, the challenges of improving the RNAi strategy for sustainable Bt cotton are still needed to be combatted [159].

### 6.5. Genome Editing

CRISPR/Cas9 technology has been demonstrated to be a promising approach to amend the genome. It significantly facilitates functional studies of both model and non-model species. This system has been used as a precise genome-editing system for various pests including *Coleoptera*, *Diptera*, *Hemipitera,* and *Lepidoptera*. CRISPR/Cas9-mediated knockout of the *Lepidoptera* olfactory receptor co-receptor *Orco* gene caused defects in plant-odor and sex-pheromone olfactory detection in homozygous individuals of the pests [160]. Genome editing of *Wnt-1*, a gene well known for its role in the early body planning in the Pine Caterpillar Moth, *Dendrolimus punctatus*, led to high embryonic mortality [161]. These results demonstrate that CRISPR/Cas9 is a simple and highly efficient technique in the development of novel pest-control strategies.

### 6.6. Bio-Control Agents

Employment of ecological friendly biopesticides particular to the mark organisms provides a promising alternative approach for pest control [162]. Biopesticides are consequential of plants and animals with active modules of microbial agents that include bacteria, virus, fungi, and algae [163]. *Spodoptera littoralis* is a polyphagous organism that assaults cotton, feeding on leaves, flower buds, fruiting points, and bolls. Damages associated with *S. littoralis* are severe in North Africa and Egypt. In a recent report, the spore suspensions of entomopathogenic fungi *Curvularia lunata, Alternaria solani*, and *A. alternata* exhibited promising controlling effects against *S. littoralis* with a mortality of 60%, 40%, and 33.3%, respectively [164]. Release of *Rhynocoris longifrons*, a generalist pillager of many cotton insect pests, in cotton fields was accomplished to reduce the population of *H. armigera* (50%), *P. solenopsis* (28%), *D. cingulatus* (18.8%), and *A. gossypii* (11.8%) during the rain-fed condition [165]. The release of natural enemies, *Chrysoperla carnea* and *Trichogramma chilonis*, incorporated with the use of artificial food sprays, consisting of different food attractants such as protein hydrolysate, sugar or a combination, has a great potential to encourage the population establishment of these as natural enemies. Subsequently, pest control was enhanced through the increased predation/parasitism percentage in thec cotton field [166].

### 6.7. Bio-Signaling and Microbial Communication

Alternative strategies other than Bt toxins would release the selective pressure of pests targeting Bt genes, and, thus, overcome the problem of the development of PRBT. In an ecosystem, plants interact dynamically with living and non-living components in their surrounding environment to elicit the adaptive and acceptable responses among different species [167]. These ecological relationships includes parasitism, symbiosis, and predation that establish complex communications among and within the species through physiological signals, pheromones, kairomones, hormones, metabolites, peptides, proteins, and RNAs [168]. Plants can sense the chemicals released by insects and prepare themselves a defense strategy through secreting anti-feedings, while the herbivorous pests may also develop mechanisms to digest such secondary metabolites or to secrete mitigants to avoid plant response to their attacks [169]. Unraveling such complex biotic relationships among different symbiotic organisms would provide tools to develop sustainable novel insect-control strategies. For example, a phytopathogenic bacteria belonging to family *Xanthomonadaceae*, produces a diffusible factor (DSF) acting as a cell-to-cell communication molecule eliciting an innate immune response in plants [170]. The expression of the enzyme responsible for producing DSF was successfully engineered in tobacco and the sweet orange for resistance against insect pests [169]. In another study, the mate recognition and localization of the grapevine pest *Scaphoideus titanus* was prevented by imitating vibrational signals with an artificial noise vibration [171]. These studies indicate that the defense system of plants against pests can be enhanced by manipulating different signals such as quorum-sensing signals through signal transduction and intracellular cascade reactions [169].

## 7. Conclusions

*G. hirsutum* is a major species for cotton production worldwide. Chemical pesticides have been used to prevent severity of insect pests and to enhance cotton yield all over the world. The adoption of genetically modified Bt cotton has presented several benefits, including reducing the load of insecticides into the cotton field, bringing better control of target pests, improving yield potential and stability, and increasing biodiversity of the non-target insects. However, it also faces some special challenges, including that the efficacy varies in different regions due to different climatic, ecological and management conditions, insect diversity, and that the target insect pests have evolved resistance against Bt cotton, which has imposed a major threat to the budget of cotton-manufacturing countries. In order to prevent or control this problem, the following approaches are recommended: (1) Novel genetic strategies together with integrated management of PRBT evolution such as a dose/refuge strategy, release of sterile insects in the field, and pyramiding of different toxin genes or genes with different pest-control mechanisms should be taken into consideration for future developments. (2) Other controlling tactics including new resources of toxin-producing genes or techniques such as RNAi and CRISPR/Cas9 are also highly recommended to overcome this insect resistance problem. (3) To succeed in improving cotton production worldwide, it is necessary to coordinate efforts from all participants around the cotton-production industry including researchers, farmers, and technicians.

In summary, cotton has become susceptible to many insect pests, and it depends on novel biotechnological solutions to prevent the losses incurred by these pests. Biotechnology solutions provide an eco-friendly system for the market of global cotton production. With the development of cotton genome sequencing and genome-editing technologies, it will bring new opportunities for cotton-pest-control strategies that shall radically change current practices to reduce losses and benefit the environment. In short, this task is still not fully completed on a global scale.

## Figures and Tables

**Table 1:** Factors affecting the expression of *cry* gene in transgenic plants.

Factors	Effects	References
Plant age	Expression of *cry* gene decreases gradually during plant development and reaches minimum at 150 days after sowing	[134,135]
Temperature	31–35 °C is the best range temperature for full expression of *cry1Ac* toxin gene in transgenic cotton	[66]
Plant parts	Leaves have high expression level, followed by bolls and seed	[134,135,136]
Humidity	High humidity induces less *cry*-gene expression	[57]
Genotype	Expression of *cry* gene varies with genotype of recipient plant	[96,128,134,135,136]
Drought	The water-deficit condition in leaf is the main cause of less Bt-toxin expression in leaf tissues	[57,137]
Promoter type	Decreased expression of *cry* gene with plant age is due to reduction in promoter activity	[134]

## Data Availability

The data presented in this study are available in article.
